# Ginsenoside CK Inhibits TGF-*β*-Induced Epithelial-Mesenchymal Transition in A549 Cell via SIRT1

**DOI:** 10.1155/2021/9140191

**Published:** 2021-12-12

**Authors:** Mingyang Sun, Xuefeng Zhuang, Guangfu Lv, Zhe Lin, Xiaowei Huang, Jiarui Zhao, He Lin, Yuchen Wang

**Affiliations:** Department of Pharmacology, School of Pharmaceutical Sciences, Changchun University of Chinese Medicine, Changchun, Jilin 130117, China

## Abstract

Ginsenoside CK is the main metabolite of protopanaxadiol saponins in intestinal bacteria. Previous studies have shown that ginsenoside CK can affect many aspects of tumor development through a variety of mechanisms. However, few studies have reported the antimetastatic effects of ginsenoside CK in non-small-cell lung cancer (NSCLC). In this study, we explored the effect of ginsenoside CK on epithelial-mesenchymal transition (EMT) induced by TGF-*β* in A549 cells and the potential molecular mechanisms. Our data showed that ginsenoside CK effectively prevented TGF-*β*-induced EMT, as indicated by the upregulation of E-cadherin and downregulation of vimentin. Furthermore, ginsenoside CK inhibited the metastatic ability of A549 cells in the tail vein lung metastasis model of nude mice. Additionally, ginsenoside CK decreased the expression of silent information regulator 2 homolog 1 (SIRT1) in the inhibition of EMT induced by TGF-*β*. Moreover, the antimetastatic effect of ginsenoside CK was reversed by SIRT1 overexpression. Generally, our results indicated the antimetastatic effect and underlying mechanism of ginsenoside CK on TGF-*β*-induced EMT in A549 cells, suggesting that ginsenoside CK can be used as an effective antineoplastic agent.

## 1. Introduction

According to statistics, lung cancer was one of the top three cancers worldwide in 2018 [1]. Moreover, lung cancer has the highest morbidity and mortality rates, with an overall 5-year survival rate of 15% [2, 3]. Among all types of lung cancer, non-small-cell lung cancer (NSCLC) accounts for more than 85% of all lung cancer patients. Clinical data showed that approximately 90% of lung cancer patients die due to tumor metastasis [4, 5].

Transforming growth factor-*β* (TGF-*β*) is an important growth inhibitor that can inhibit the growth of most normal cells; it also plays an important role in regulating various cell behaviors, including invasion, migration, differentiation, and changes in the microenvironment [6]. TGF-*β* mainly includes TGF-*β*1, TGF-*β*2, and TGF-*β*3 in mammals. TGF-*β*1 not only promotes tumor migration and invasion but also inhibits tumor cell growth [6, 7]. Overexpression of TGF-*β*1 in advanced stages of lung cancer can promote tumor growth and metastasis [8]. In addition, for patients with NSCLC, high TGF-*β*1 expression is an important prognostic parameter after operative treatment.

Recent studies have shown that silent information regulator 2 homolog 1 (SIRT1), a class 3 histone deacetylase, is involved in regulating the invasion and metastasis of various tumor cells [9–11]. Specifically, SIRT1 is overexpressed in NSCLC tissues, as well as in NSCLC cell lines such as A549, H1299, and H460 [12, 13]. Furthermore, SIRT1 is an important marker of poor prognosis [12]; SIRT1 is associated with poor clinicopathological manifestations, including tumor size, lymph node metastasis, and advanced tumor invasion [14]. SIRT1 overexpression downregulates E-cadherin and upregulates N-cadherin. It is worth noting that TGF-*β* can upregulate the expression of SIRT1, and SIRT1 overexpression can downregulate E-cadherin and upregulate N-cadherin, promoting the EMT of A549 cells (a NSCLC cell line), suggesting that SIRT1 may be an important protein in the TGF-*β* signaling pathway [13]. Due to the potential role of SIRT1 in migration, invasion, and metastasis, the role of SIRT1 in EMT has also received significant attention in NSCLC.

Panax ginseng has been used as a medicinal plant in Asian regions for thousands of years, and modern pharmacological research has shown that Panax ginseng has anti-inflammatory, anticancer, and other pharmacological activities [15, 16]. Ginsenosides have been considered the main bioactive ingredients, and recent studies have reported that ginsenoside can inhibit many aspects of tumor development through a variety of mechanisms, such as direct cytotoxicity, growth inhibitory effects against tumor cells, and tumor cell metastasis [17–20]. Ginsenoside CK is the final metabolite of protopanaxadiol saponins (PPD) [21, 22]. As metabolized by intestinal bacteria, ginsenoside CK has better absorption and diverse bioactive activities *in vivo* [18]. However, there are few reports that ginsenoside CK inhibits lung cancer EMT and tumor cell metastasis. Ginseng metabolites have been largely described in EMT, and other reports also mentioned the ability of ginsenoside CK to inhibit EMT of tumor cells, including breast cancer [23] and liver cancer [24]. Thus, it is reasonable to propose that ginsenoside CK might show some degree of inhibitory effects in regulating the EMT of lung cancer cells.

In this study, we evaluated the effect of ginsenoside CK on TGF-*β*-induced EMT. Our data indicated that ginsenoside CK suppressed TGF-*β*-induced EMT, migration, and invasion *in vitro* and suppressed A549 cell metastasis *in vivo*. Our previous research revealed that the mechanism by which PPD suppresses Ang II-induced EMT is related to the decrease in SIRT1 in A549 cells [25]. Therefore, we investigated the changes in SIRT1 expression during the pharmacological effects of ginsenoside CK. Our data show that the downregulation of SIRT1 is involved in the reversion of EMT primed by TGF-*β* in A549 cells treated with ginsenoside CK.

## 2. Materials and Methods

### 2.1. Cell Culture and Reagents

Ginsenoside CK was provided by Chengdu Desite Co., Ltd. (Chengdu, Sichuan, China). A549 cells were obtained from Procell Life Science &Technology Co., Ltd. (Wuhan, Hubei, China) and were incubated in RPMI-1640 medium (Hyclone, Logan, UT, USA) with 10% FBS, 100 units/mL penicillin, and 100 mg/mL streptomycin. The cells were maintained at 37°C in a humidified atmosphere containing 5% CO_2_. FBS was purchased from Biological Industries (Kibbutz Beit HaEmek, Israel). TGF-*β* was purchased from PeproTech, Inc. (Suzhou, Jiangsu, China). BCA protein assay reagent kits and DAPI were purchased from Beijing Solarbio Science & Technology Co., Ltd. (Beijing, China). Antibodies against E-cadherin, vimentin, SIRT1, and secondary antibodies were obtained from Proteintech Group, Inc. (Chicago, IL, USA).

### 2.2. Wound Healing Assay

A549 cells were seeded into a 6-well plate at a cell concentration of 3 × 10^5^ cells·mL^−1^. Cells were cultured for 24 h in an incubator with cells forming single-layer fusion scratches. A sterile 10 *μ*L pipette tip was used to scratch through each hole perpendicular to the horizontal line. To remove suspended cells, the cells were washed with serum-free medium and then treated with 1.5 *μ*g·mL^−1^ or 3 *μ*g·mL^−1^ ginsenoside CK and 10 ng·mL^−1^ TGF-*β* in serum-free medium for 24 h. Images were captured, and the scratch width was recorded at 0 h and 24 h using an inverted microscope. Quantitation was performed using ImageJ software (version 1.5.0.26; National Institutes of Health, Bethesda, MD, USA). The experiment was performed three times.

### 2.3. Transwell Assay

For the Transwell invasion assay, A549 cells were resuspended in serum-free medium at a cell concentration of 3 × 10^5^ cells·mL^−1^ and seeded onto the upper surfaces of Matrigel-coated Transwell chambers (BD Biosciences, San Jose, CA, USA). The bottom of the chambers was filled with 600 *μ*L of 10% FBS medium containing 1.5 *μ*g/mL^−1^ or 3 *μ*g·mL^−1^ ginsenoside CK. After 4 h of incubation, TGF-*β* was added to the medium at the bottom to a final concentration of 10 ng·mL^−1^. After 24 h incubation, cells invading the lower surface of the chamber were fixed with 4% paraformaldehyde and stained with crystal violet solution. Five fields were randomly captured, and the cells were counted under an inverted microscope. The experiment was performed three times.

### 2.4. Quantitative Real-Time PCR

Total RNA was isolated using TRIzol reagent (Invitrogen, Carlsbad, CA, USA). The absorbance of the RNA at 260 nm and 280 nm was measured using a UV spectrophotometer and then reverse-transcribed into cDNA using a TransScript® Green Two-Step qRT-PCR SuperMix kit (TransGen Biotech, China), according to the manufacturer's instructions. qRT-PCR was conducted using the TransScript Green Two-Step qRT-PCR SuperMix (TransGen Biotech, China). The amount of mRNA was normalized to that of GAPDH. The primer sequences are listed in Supplementary Table 1. Relative fold changes were determined using the 2^–*ΔΔ*CT^ method.

### 2.5. Western Blotting

As described previously [25], cells were lysed in cell lysis buffer (Beyotime Biotechnology) on ice. Protein samples were mixed with sample loading buffer and boiled in water at 100°C for 5 min to denature the proteins. Proteins were then separated using SDS-PAGE and transferred onto a PVDF membrane (Millipore, Burlington, MA, USA). The membranes were incubated with primary antibodies against E-cadherin, vimentin, SIRT1 (Abcam, Cambridge, United Kingdom), and GAPDH (ZSGB Biotechnology Co., Ltd., Beijing, China) at a 1 : 1,000 dilution at 4°C overnight. Protein expression was analyzed by binding with a secondary antibody using a BeyoECL Plus enhanced chemiluminescence kit. ImageJ software was used for analysis.

### 2.6. Immunofluorescence Staining

Cells were cultured on glass-bottom cell culture dishes and incubated with TGF-*β* and ginsenoside CK for 24 h. The cells were then harvested and fixed with paraformaldehyde and precooled methanol for 15 min, followed by washing with PBS three times. The cells were then permeabilized with 0.3% Triton X-100 for 15 min and blocked with 5% goat serum for 1 h at room temperature. Cells were incubated with anti-E-cadherin and anti-SIRT1 antibody (Abcam, Cambridge, United Kingdom, 1 : 200), diluted in 5% goat serum, and incubated overnight at 4°C. After washing with PBS, cells were incubated with Cy3-conjugated, FITC-conjugated secondary antibody (Beyotime Biotechnology) for 1 h at room temperature away from light. After washing away excess antibodies, the cells were sequentially stained with DAPI for nuclear staining, and the samples were imaged using a fluorescence microscope.

### 2.7. Tail Vein Injection Lung Cancer Models

As described previously [26, 27], male BALB/c nude mice (Beijing HFK Bio-Technology, Beijing, China) were injected via the tail vein with A549 cells (1 × 10^6^ cell/mouse). The mice from each group were anesthetized by exposure to 3% isoflurane and intraperitoneally injected with XenoLight D-Luciferin Potassium Salt (150 mg·kg^−1^) every 7 days for up to 21 days. After 15 min, the photons emitted from the tumor were monitored using a Bruker In Vivo Xtreme (Bruker, Billerica, MA, USA). Nude mice were orally administered 10 mg·kg^−1^ and 20 mg·kg^−1^ ginsenoside CK for 3 weeks. Nude mice were sacrificed at the end of the experiment, after which their lungs were removed and fixed in 10% formalin.

The experimental design was in strict accordance with the principles and guidelines recommended by the Committee for the Care and Use of Laboratory Animals of Changchun University of Chinese Medicine (Changchun, China) and approved by the Ethics Committee of Changchun University of Chinese Medicine. Male nude mice were provided by Beijing HFK Bio-Technology (Beijing, China).

### 2.8. Statistical Analysis

All data are expressed as the mean ± SD of at least three individual experiments. Statistical analyses were performed using SPSS Statistical 24 Software. Two-way ANOVA and Bonferroni post hoc analyses were used to calculate the statistical differences between multiple groups. For all statistical tests, a *P* value < 0.05 was considered statistically significant.

## 3. Results

### 3.1. Ginsenoside CK Inhibits TGF-*β*-Induced A549 Cell EMT

**Figure 1:**
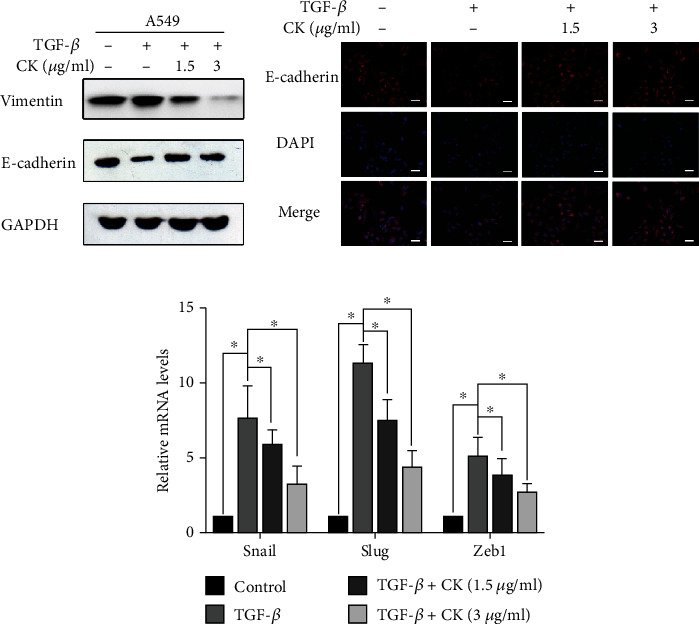
Effect of ginsenoside CK on TGF-*β*-induced EMT in A549 cells. (a) A549 cells were treated with TGF-*β*, with or without 1.5 *μ*g·mL^−1^ or 3 *μ*g·mL^−1^ ginsenoside CK for 24 h, and the protein expression of vimentin and E-cadherin was determined by Western blotting (a) and immunofluorescence staining (b). (c) The mRNA levels of EMT-related transcription factors, including Snail, Slug, and Zeb1, were measured using qRT-PCR. All scale bars represent 50 *μ*m. Error bar, SD of three independent experiments. ^∗^*P* < 0.05.

Various extracellular stimuli and intracellular signal transduction pathways are involved in the induction of EMT. TGF-*β* is one of the most representative cytokines that plays an important role in EMT induction in different tissues and participates in the regulation of EMT and tumor cell migration [28]. As shown in Figures 1(a) and 1(b), TGF-*β* increased vimentin protein expression and reduced E-cadherin protein expression, which are markers of EMT. According to the MTT results (Supplementary Figure 1), we choose 1.5 *μ*g/mL and 3 *μ*g/mL ginsenoside CK for subsequent EMT and transfer-related experiments. Ginsenoside CK significantly inhibited TGF-*β*-induced changes in vimentin and E-cadherin expressions. Furthermore, we investigated the mRNA levels of EMT-related transcription factors Snail, Slug, and Zeb1 to evaluate the anti-EMT effects of ginsenoside CK. As shown in Figure 1(c), ginsenoside CK suppressed TGF-*β*-induced EMT-related transcription factors. Similar results were observed in H1299 cells, and ginsenoside CK reversed the changes in vimentin and E-cadherin expression (Supplementary Figure 2). These data indicate that ginsenoside CK treatment inhibited TGF-*β*-induced EMT in A549 cells.

### 3.2. Ginsenoside CK Inhibits TGF-*β*-Induced Migration and Invasion of A549 Cells

**Figure 2:**
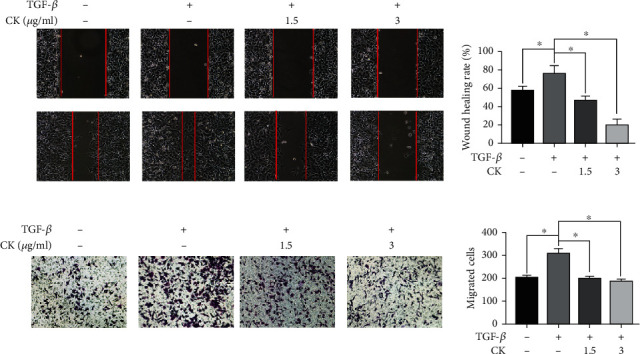
Effect of ginsenoside CK on the migration and invasion abilities of A549 cells with TGF-*β*1 pretreatment. A549 cells were treated with 10 ng/mL TGF-*β*1 for 24 h, with or without 1.5 *μ*g·mL^−1^ or 3 *μ*g·mL^−1^ ginsenoside CK and subjected to (a) wound healing assay and (b) the Matrigel-coated Transwell assays to assess tumor cell invasion. Error bar, SD of three independent experiments. ^∗^*P* < 0.05.

EMT is one of the keys to the progression of cell cancer metastasis, and the most significant change in tumor cells is the enhancement of the migration and invasion abilities [29]. To evaluate the effects of ginsenoside CK on migration and invasion in A549 cells, wound healing and Transwell assays were carried out. As shown in Figures 2(a) and 2(b), TGF-*β* treatment significantly increased the migration and invasion abilities of A549 cells. Ginsenoside CK treatment markedly suppressed TGF-*β*-induced migration and invasion of A549 cells (Figure 2) and H1299 cells (Supplementary Figure 2).

### 3.3. Ginsenoside CK Reduces MMP-2 and MMP-9 in A549 Cells

**Figure 3:**
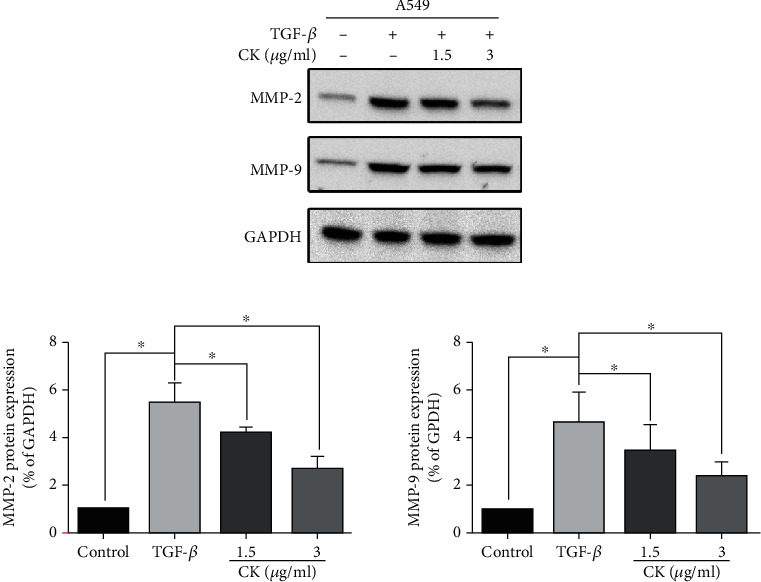
Effect of ginsenoside CK on the expression of MMP-2 and MMP-9. A549 cells were treated with TGF-*β*, with or without 1.5 *μ*g·mL^−1^ or 3 *μ*g·mL^−1^ ginsenoside CK for 24 h, and (a) the protein expression of MMP-2 and MMP-9 was determined by Western blotting. (b) The expressions of MMP-2 and MMP-9 were quantified by Gray analysis. Error bar, SD of three independent experiments. ^∗^*P* < 0.05.

Transwell assay results suggested that ginsenoside CK has the potential to resist invasion. As MMP-2 and MMP-9 are believed to play a critical role in tumor invasiveness, we determined the expression of these proteinases using Western blotting. As shown in Figure 3, the expression of MMP-2 and MMP-9 was suppressed by ginsenoside CK in a dose-dependent manner.

### 3.4. Ginsenoside CK Inhibits A549 Cell Metastasis *In Vivo*

**Figure 4:**
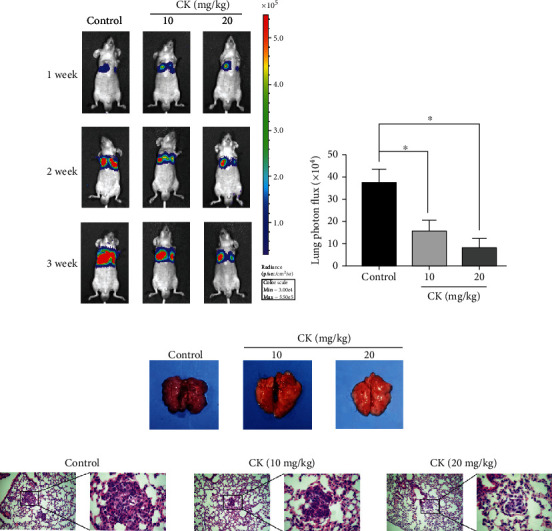
Effects of ginsenoside CK on lung metastasis *in vivo*. Female BALB/c nude mice were injected via the tail vein with A549 cells and were orally administered with 10 mg·kg^−1^ or 20 mg·kg^−1^ ginsenoside CK for 3 weeks. (a) Bioluminescence imaging of the mice after injection at weeks 1, 2, and 3. Bioluminescence of the lung is shown. (b) Mean bioluminescence of the lung in tail vein pulmonary metastasis mice at 3 weeks. (c) Representative images of lung metastatic nodules. (d) Representative pictures of HE staining of the lung tissue are shown (magnification, (A) ×100 and (B ×400). ^∗^*P* < 0.05.

**Figure 5:**
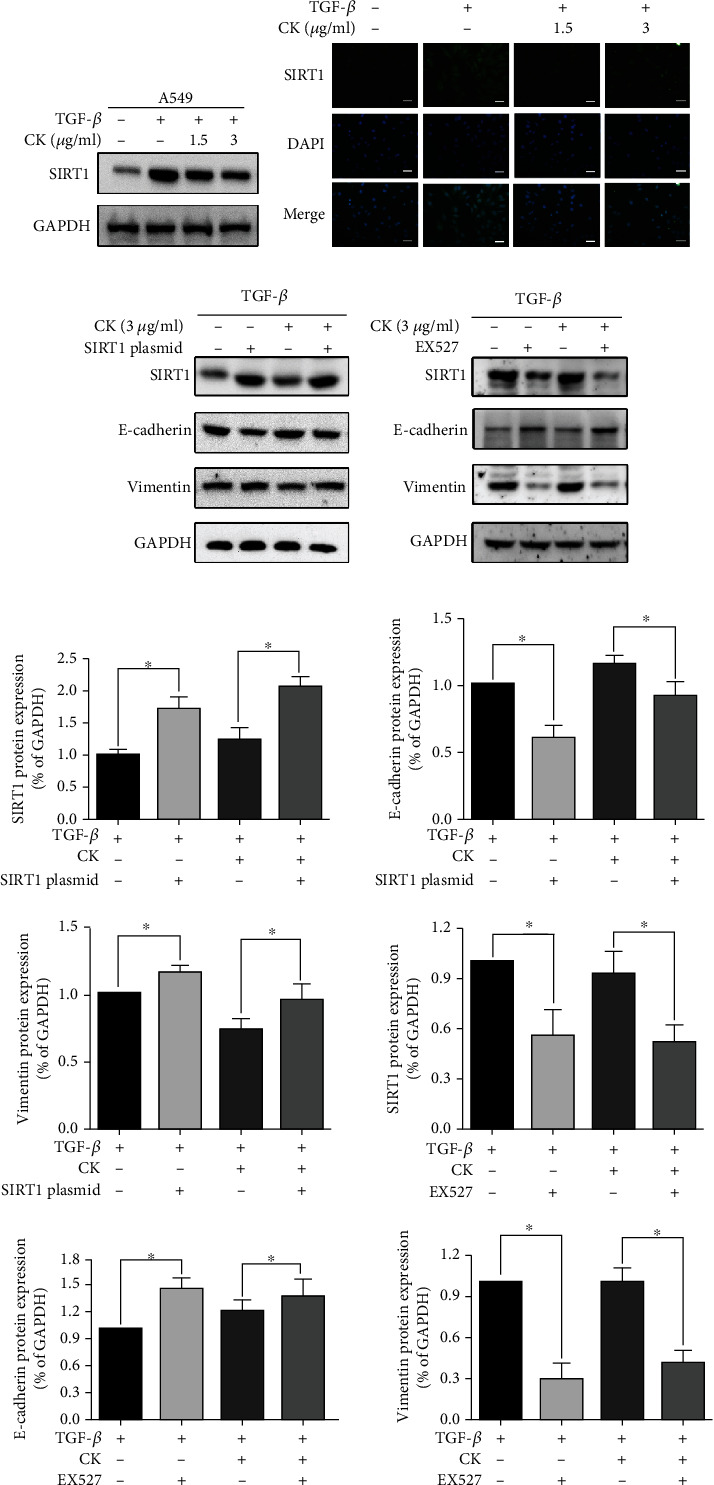
Involvement of SIRT1 in TGF-*β*-induced EMT inhibited by ginsenoside CK. A549 cells were treated with TGF-*β*, with or without 1.5 *μ*g·mL^−1^ or 3 *μ*g·mL^−^1 ginsenoside CK for 24 h, and the protein expression of SIRT1 was determined by Western blotting (a) and immunofluorescence staining (b). (c) A549 cells were transfected with SIRT1 plasmid with or without 1.5 *μ*g·mL^−1^ or 3 *μ*g·mL^−1^ ginsenoside CK for 24 h. Representative Western blotting images for vimentin and E-cadherin in A549 cells are shown. (d) Cells were treated with the SIRT1 inhibitor EX-527 and then further incubated in the presence of ginsenoside CK for 24 h. The expression of E-cadherin, vimentin, and SIRT1 was determined by Western blotting. (e) The expressions of SIRT1, E-cadherin, and vimentin were quantified by Gray analysis. Error bar, SD of three independent experiments. ^∗^*P* < 0.05.

To confirm the antimetastatic activity of ginsenoside CK, we established a nude mouse lung metastasis model to simulate the metastatic stage of advanced NSCLC. Based on previous studies and preliminary experiments, we chose 10 mg·kg^−1^ and 20 mg·kg^−1^ ginsenoside CK for the experiment [30]. Bioluminescence image analysis showed that ginsenoside CK decreased lung tumor formation compared to that in the control group (Figure 4(a)). Meanwhile, the fluorescence intensity of A549 cells decreased in the ginsenoside CK group (Figure 4(b)). The number of metastatic nodules per lung was substantially reduced by ginsenoside CK treatment (Figure 4(c)). Additionally, histological analysis of the lungs revealed significantly decreased multiplicity and volume of tumor nodules in the ginsenoside CK-treated mice compared to control group mice (Figure 5(d)). These results further support the antimetastatic effect of ginsenoside CK.

### 3.5. Involvement of SIRT1 in TGF-*β*-Induced A549 Cell EMT Inhibition by Ginsenoside CK

**Figure 6:**
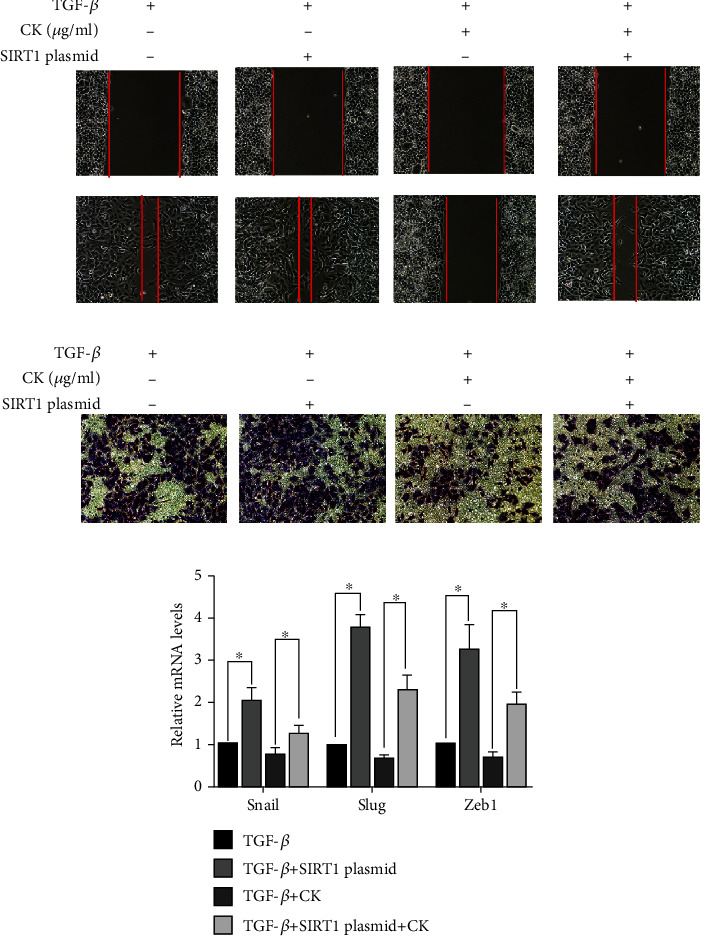
Effect of SIRT1 overexpression on the migration and invasion abilities of A549 cells with ginsenoside CK pretreatment. Cells were transfected with SIRT1 plasmid and then further incubated in the presence of ginsenoside CK for 24 h. Wound healing assay and Transwell assays assessed tumor cell migration capacity in A549 cells. The mRNA levels of Snail, Slug, and Zeb1 were measured by RT-PCR. ^∗^*P* < 0.05.

SIRT1 is a downstream molecule of TGF-*β*. We found that reducing the expression of SIRT1 is an important mechanism of PPD against invasion and metastasis [25]. Therefore, we speculate that SIRT1 also plays an important role in the process by which ginsenoside CK suppresses the invasion and metastasis of A549 cells induced by TGF-*β*. We next examined the expression of SIRT1 using Western blotting and immunofluorescence staining. Consistent with previous studies [13], our data showed that TGF-*β* increased the expression of SIRT1. Ginsenoside CK attenuated the upregulation of SIRT1 by TGF-*β* (Figures 5(a)–5(b)). We first examined whether TGF-*β*-induced EMT inhibition by PPD could be counteracted by SIRT1 upregulation. We transfected a SIRT1-expressing plasmid into A549 cells. As shown in Figures 5(c) and 6, SIRT1 overexpression markedly increased TGF-*β*-induced EMT, migration, and invasion, even in the presence of ginsenoside CK. Next, we downregulated SIRT1 expression using EX-527, a SIRT1 inhibitor. Figure 5(d) shows that EX-527 acted synergistically with ginsenoside CK to inhibit TGF-*β*-induced EMT, indicating that SIRT1 is a key molecule of ginsenoside CK that antagonizes TGF-*β*.

## 4. Discussion

Ginsenoside is an antitumor traditional Chinese medicine that has attracted much attention. The antitumor effects of ginsenosides are multifaceted and multitargeted. Ginsenoside CK is a typical ginsenoside [31]. Ginsenoside CK has been reported to be cytotoxic to human and animal tumor cell lines and inhibits their proliferation. It has potential cytotoxicity in mouse highly metastatic melanoma, human liver cancer, human myeloid leukemia, and human highly metastatic lung cancer cells [32]. However, current studies have focused on cytotoxicity, while few studies have reported the inhibitory activity of ginsenoside CK against EMT in A549 cells.

In this study, we investigated the potential effect and mechanism of ginsenoside CK on the inhibition of EMT, invasion, and metastasis *in vitro* and *in vivo*. First, our data demonstrated that noncytotoxic concentrations of ginsenoside CK inhibited EMT biomarker changes in response to TGF-*β* and suppressed the migration of NSCLC A549 cells after TGF-*β* stimulation (Figure 1). In addition, we performed an animal experiment to verify the inhibitory effect of ginsenoside CK on tumor metastasis in A549 cells. Next, we attempted to identify the changes in SIRT1 in ginsenoside CK, which inhibits EMT. Previously, we reported that PPD, a ginseng metabolite, suppressed Ang II-induced A549 cell EMT, invasion, and metastasis by reducing SIRT1 *in vitro* and *in vivo* [25]. Thus, SIRT1 represents a potential target of ginsenoside for inhibiting EMT. The results revealed that ginsenoside CK reduced SIRT1 protein expression *in vitro*. Our *in vitro* and *in vivo* experiments indicate that these effects of ginsenoside CK were closely related to the suppression of SIRT1.

EMT, a cellular process that transforms cells from epithelial to motional mesenchymal phenotype, promotes invasion and metastasis of cancer cells and is a prognostic marker in tumor development [33]. Changes in the expression of specific transcription factors are thought to be major steps in EMT, and changes in the expression of target genes can lead to cytoskeletal reconstruction. For example, cytokeratin is converted into vimentin [34]. In addition, specific proteins associated with cell-cell adhesion and cell motility are converted into mesenchymal-specific markers [35–37]. EMT is regulated by a variety of extracellular stimulation and intracellular signal transduction pathways, such as the Wnt pathway, growth factor receptor tyrosine kinases, and other signaling pathways, among which the TGF-*β* pathway is the most representative [38]. TGF-*β* signaling has been shown to play an important role in the EMT. In fact, adding TGF-*β* to epithelial cells in culture is a convenient way to induce EMT in various epithelial cells. As previously reported, our results showed that E-cadherin downregulation, a marker of mesenchymal cells such as E-cadherin, vimentin, MMP-2, and MMP-9 were also upregulated in A549 cells treated with TGF-*β*. In this study, we found that ginsenoside CK inhibited TGF-*β*-induced migration and invasion of A549 cells and reduced the levels of EMT-associated proteins, transcription factors, and matrix metalloproteinases. Moreover, we used a nude mouse lung metastasis model, as few studies have reported the effects of ginsenoside CK in the model. Ginsenoside CK significantly suppressed the pleural dissemination of cancer cells compared to the control group.

SIRT1 belongs to the class III histone deacetylase family. Recent studies have confirmed that regulation of SIRT1 expression can affect the malignancy of various cancer cells *in vitro* and *in vivo* [39–41]. Because SIRT1 affects tumor metastasis and malignancy, SIRT1 is considered to be a key regulator of EMT [42, 43]. However, the impact of SIRT1 on EMT varies in various types of cancers. It has been reported that SIRT1 is overexpressed in pancreatic cancer tissues and is a promoter of the occurrence and metastasis of pancreatic cancer [44]; however, SIRT1 has the opposite effect in oral squamous cell carcinoma, which inhibits EMT through the SIRT1/Smad4/MMP7 pathway [45]. SIRT1 is a promoter of EMT in NSCLC SIRT1 is overexpressed in NSCLC cell lines such as A549, H460, and H1299, and overexpression of SIRT1 can downregulate E-cadherin and upregulate N-cadherin. It is noteworthy that TGF-*β* is thought to be a regulator of SIRT1 as SIRT1 expression increases in TGF-*β*-induced EMT [13]. In this study, we found that ginsenoside CK suppressed SIRT1 levels in A549 and H1299 cells. Similarly, EX527, an inhibitor of SIRT1, also reduced cell migration and invasiveness. In addition, SIRT1 plasmid transfection in A549 cells partially reversed the effect of ginsenoside CK. This suggests that ginsenoside CK plays a critical role in inhibiting SIRT1, which is an important mediator of EMT.

This study had several limitations. First, we did not examine the correlation between SIRT1 and TGF-*β* signaling. Although SIRT1 is known to be a downstream molecule of TGF-*β*, the mechanism by which TGF-*β* activates SIRT1 remains unclear. Second, although the animal experiment was performed to verify the *in vivo* effects of ginsenoside CK, an animal experiment has not been performed with stimulation of TGF-*β* with an osmotic pump, and the interference effects of ginsenoside CK on TGF-*β* in vivo were not examined.

## 5. Conclusions

In this study, we found that ginsenoside CK inhibited migration and invasion *in vitro* and metastasis *in vivo*. Furthermore, ginsenoside CK suppressed TGF-*β*-induced EMT in A549 cells. It is worth noting that inhibition of SIRT1 could be a pivotal mechanism in the inhibitory effect of ginsenoside CK on EMT. Accordingly, this study provides a new idea and theoretical basis for the development of ginsenoside CK as an antitumor drug.

## Data Availability

The datasets generated during and analyzed during the current study are available from the corresponding author on reasonable request.

## Supplementary Materials

Supplementary Materials Supplementary Table 1: primer sequences for gene expression analysis by RT-PCR in A549 cell. Supplementary Figure 1: effects of ginsenoside CK on the cell viability in A549 cells. Supplementary Figure 2: effect of ginsenoside CK on EMT, migration, and invasion abilities of H1299 cells with TGF-*β*1 pretreatment.
