# Supplementary material for: Ginsenoside CK Inhibits TGF-β-Induced Epithelial-Mesenchymal Transition in A549 Cell via SIRT1
Source: Biomed Res Int. 2021 Dec 12;2021:9140191. doi: 10.1155/2021/9140191 (PMC8684819; doi:10.1155/2021/9140191)
Supplement: Supplementary Materials — Supplementary Table 1: primer sequences for gene expression analysis by RT-PCR in A549 cell. Supplementary Figure 1: effects of ginsenoside CK on the cell viability in A549 cells. Supplementary Figure 2: effect of ginsenoside CK on EMT, migration, and invasion abilities of H1299 cells with TGF-β1 pretreatment. [file 9140191.f1.docx]

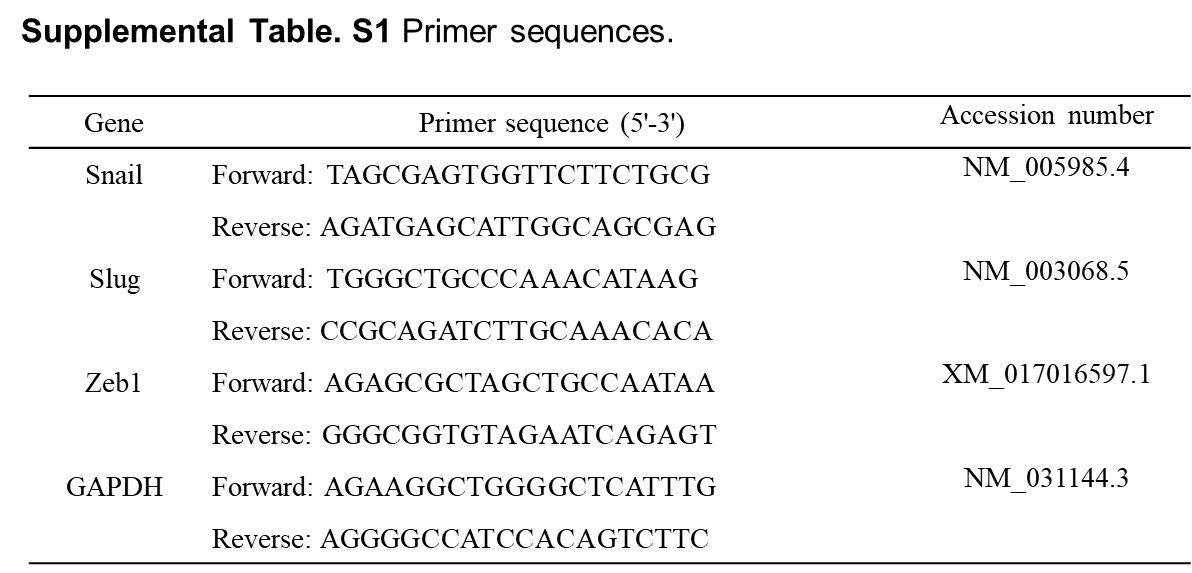


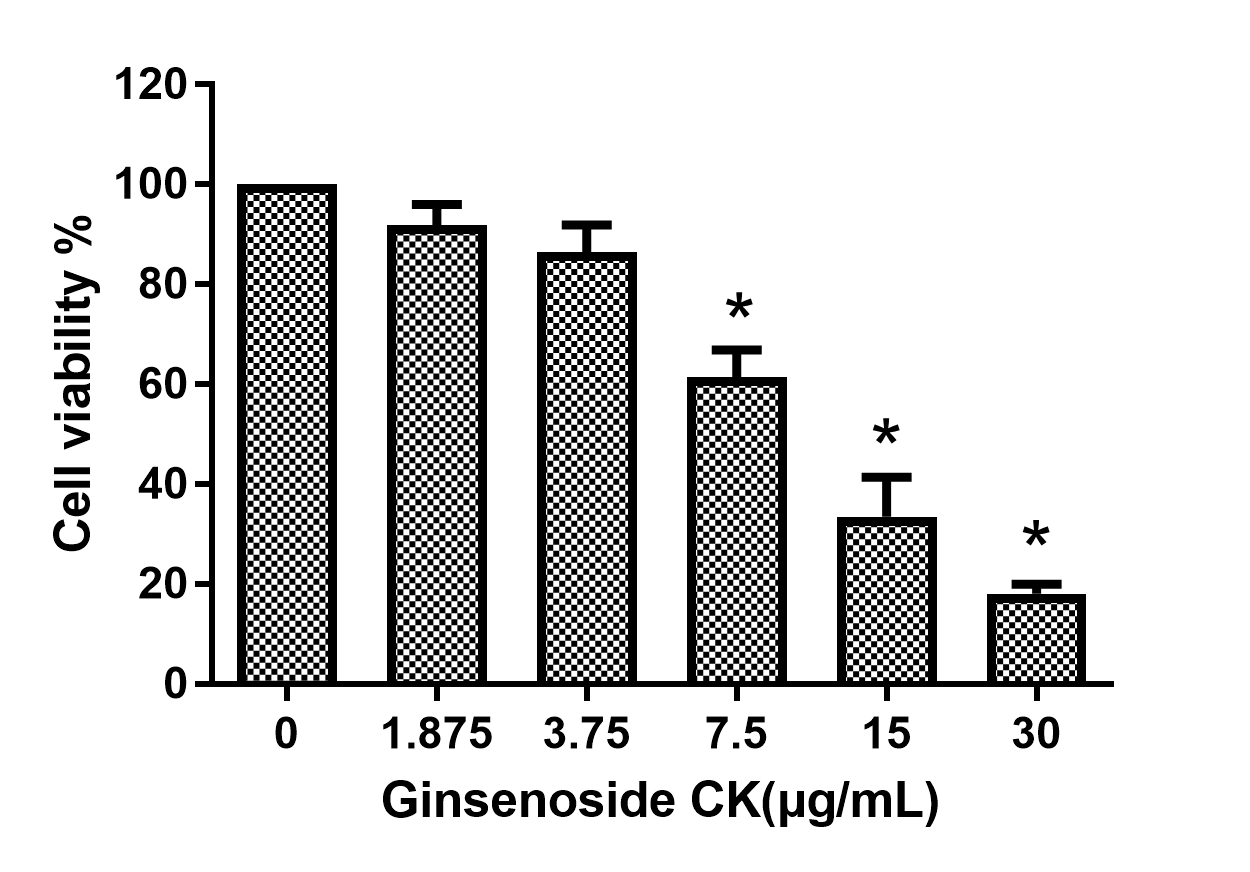


Supplementary Figure1. Effects of Ginsenoside CK on the cell viability in A549 cells. Data are expressed as the means ± SD; *p<0.05.


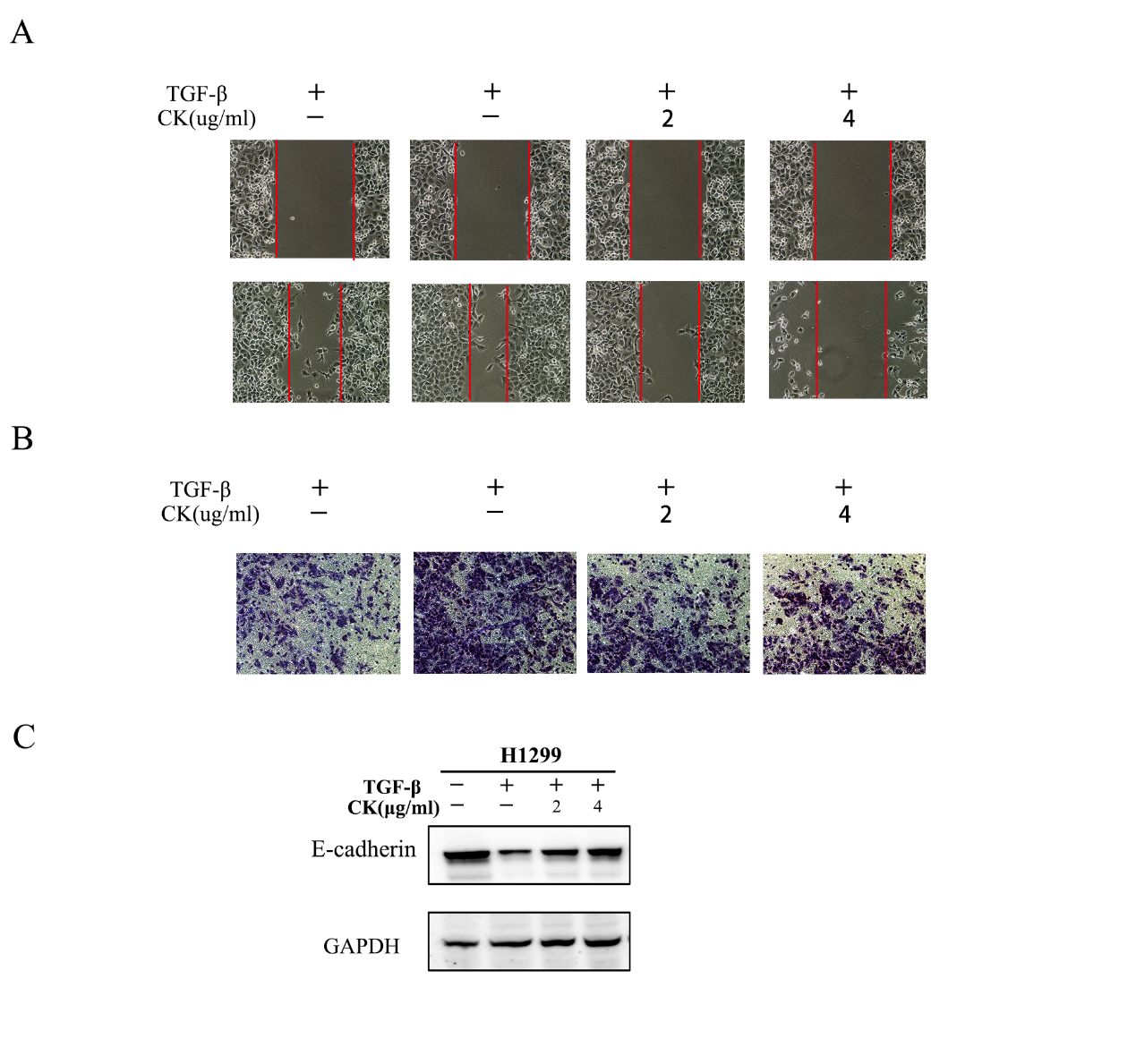


Supplementary Figure2. Effect of Ginsenoside CK on EMT, the migration and invasion ability of H1299 cells with TGF-β pretreatment.
